# How therapists and patients need to develop a clinical feedback system after 18 months of use in a practice-research network: a qualitative study

**DOI:** 10.1186/s13033-021-00465-z

**Published:** 2021-05-11

**Authors:** Christian Moltu, Andrew A. McAleavey, Marianne M. Helleseth, Geir Helge Møller, Sam S. Nordberg

**Affiliations:** 1 Department of Psychiatry, District General Hospital of Førde, Førde, Norway; 2 Department of Health and Caring Sciences, Western Norway University of Applied Sciences, Førde, Norway; 3 Weill Cornell Medical College, New York, NY USA

**Keywords:** Routine Outcome Monitoring, Clinical Feedback, Implementation, Action Research

## Abstract

**Background:**

A personalized computer-adaptive system for clinical feedback and routine outcome monitoring in mental health, Norse Feedback aims to bridge the needs for standardized and idiographic measures in ordinary practice.

**Methods:**

Item response theory analyses of completed treatment processes (n = 800) informed a qualitative study comprising individual in-depth interviews and focus groups with patients (n = 9) and clinicians (n = 10). The research question was: How do clinicians and patients contribute to developing a clinical feedback system in a continuous process aimed at refining its clinical usefulness?

**Results:**

We conducted thematic analyses and found five themes: 1. Added clinical needs, 2. Needs for re-organizing the clinician report, 3.Needs for differentiation of clinical content, 4. User-interface needs, and 5. Item level suggestions.

**Conclusion:**

In this article, we detail resulting needs for continuous adaptation to practice, and discuss implications of the concrete experiences with the Norse action research program for the larger field of ROM/CFS implementation.

## Background

While researchers have advocated for using Routine Outcome Monitoring (ROM) and Clinical Feedback Systems (CFS) over the past two decades [1–4], therapists have proven more reluctant to taking up the practice in their clinical work [5–7]. Several meta-analyses have concluded that ROM/CFSs can produce beneficial effects [1, 3], but a recent independent review found significant variability in the effects on patient outcomes produced by ROM/CFSs [8], concluding that evidence is still insufficient to advocate their general use. Importantly, what is meant by ROM/CFS in clinical practice is quite complex: A scoping review reported concerning diversity in how ROM/CFS is understood, used and implemented [9], and found the best results in settings where ROM/CFSs were structurally integrated in clinical processes. Clearly, concerns that ROM/CFSs risk becoming administrative exercises rather than helpful clinical tools [10] still seem relevant, and a basic premise from ROM/CFSs inception warrants repetition: For such tools to be beneficial, they need to be developed from and support the clinical needs of patients and clinicians [4, 11, 12].

ROM is the practice of collecting patient reported data on mental health status, symptoms and development throughout treatment in naturalistic settings [13]. This can be done for several purposes [14, 15] through standardized measures or by using idiographic items generated by the patients themselves [16]. CFS in one specification of ROM usage. In CFS, information provided by the patient through ROM is made immediately available and actionable in the sessions to support clinical conversations, establish and evaluate treatment focus and goals [17]. A qualitative meta-analysis of 16 patient perspective studies reported that patients appreciated the CFS when it captured the complexity of their treatment processes and was used for collaborative practice [18]. Recent studies exploring how to implement and use patient-reported data well in the clinical encounter [17, 19–21] suggested that understanding CFS as a clinical process that therapists need to become safe with can be a fruitful conceptualization.

While ROM/CFSs show potentials for improving practice, implementation issues, technical issues, top-down issues leading to recipient resistance, clinicians’ and patients’ needs for personalized approaches mirroring their specific clinical situation, and conceptual and operational diversity in the ROM/CFS field [7, 22–24] all yield interesting challenges for those who wish to develop, implement and research a ROM/CFS system that works well in the clinic [4]. One concept that addresses many of these challenges is practice-research networks (PRNs) [25, 26] in which researchers and clinicians and their patients collaborate on developing practice-based evidence and innovations, for example through participatory research methods [27]. Within this strategy, clinicians and patients are not expected to passively receive what research offers them, but rather to be active partners in defining needs, acceptability and utility in the knowledge production process. The overarching framework of action research (AR) [28] allows participants influence, in that they partner in co-designing the study, participate in data collection, and tries out the results in practice. The AR process uses scientific qualitative and quantitative methods in an iterative sequence to develop, study, and refine a particular clinical intervention [29].

Aiming to develop a ROM/CFS system that mirrored clinical needs and was useful to clinicians and patients, Norse Feedback was initiated and developed within the frame of a PRN action research program [29]. Initial studies sought to document patients’ and clinicians’ need from a feedback system [30], their understanding of the outcomes that they aimed to achieve [31] and patients’ experiences with CFSs in general [18]. From this departure point, a first iteration of Norse Feedback was developed and implemented digitally in the PRN clinics. During the first cycle we conducted several studies on implementation and clinical use [17, 19, 20, 22, 32], to inform refinement for the second cycle. Moreover, the first implementation provided rich quantitative data on Norse Feedback psychometrics [29, 33] and qualitative data from clinicians’ and patients’ experiences with the system in itself.

This article reports the results from a mixed method study of Norse Feedback’s functionality as CFS, as part of the transition from the first to the second version. After the first cycle implementation over an 18-month period, we conducted this study of clinicians’ and patients’ experiences with Norse Feedback’s functions and usefulness. Item response theory (IRT) analyses of completed treatment processes (n = 800) were used as primers for the qualitative part of the study with patients and clinicians who had in-depth experience with using Norse Feedback. The research question was: How do clinicians and patients contribute to developing a clinical feedback system in a continuous process aimed at refining its clinical usefulness?

## Method

### Procedure

The present study is a mixed method study where we first used quantitative methods to establish the function and information value of items and dimensions on the first Norse Feedback iteration, embedded in an overarching action research [28, 29] program. The quantitative results in themselves can be found elsewhere [33]. In the present study, quantitative results were solely used to inform and prime the qualitative data collection, by being shown to and discussed in the focus groups and individual interviews where qualitative data were collected.

### CFS study context: Norse Feedback

**Fig. 1:**
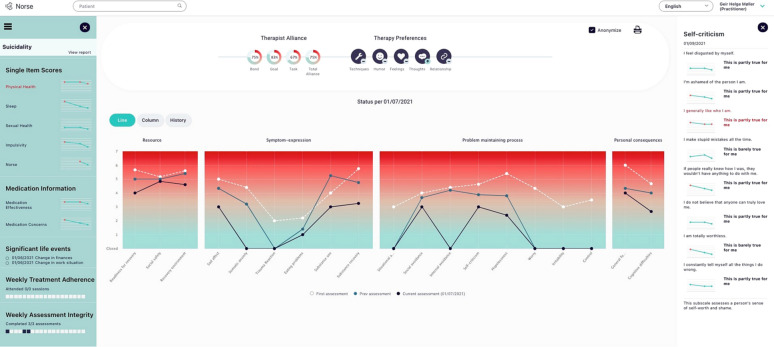
Clinician report

NF is a multidimensional ROM/CFS for mental health contexts, developed at Helse Førde Hospital Trust since 2015. It is digitally administered to patient phones over a tailored platform to allow user-friendly and data-secure deliveries. Clinicians receive clinical reports directly integrated with the hospital’s electronic health record (Fig. 1). In the first development cycle, NF started out with an intake measure comprising 90 items over 17 dimensions (Avoidance, Hopelessness, Eating concerns, Emotional distancing, Negative rumination, Hypervigilance, Perfectionism, Sad affect, Psychotic thinking, Interpersonal difficulty, Somatic anxiety, Substance abuse, Suicidal concerns, Attachment safety, Sense of belonging, General resilience, Social Functioning). Employing an empirical algorithm analysing the patients’ input in real time, continuous item personalization to the individual patient happened from the second session onwards. Dimensions that prove irrelevant for any individual patient are collapsed, and are subsequently represented only by a trigger item. In this way, the patient gets an adaptation to his or her problems and resources. The trigger item for a dimension is the item shown through IRT analyses to provide most the discriminatory information in the mild to moderate difficulty area of a dimension. If a collapsed dimension again becomes relevant during the therapeutic process, patient responses above an empirical threshold on the trigger item reopen the dimension instantaneously. From the second administration onwards, items pertaining to therapeutic alliance and patients’ experienced needs in therapy open. The different dimensions potentially measured by Norse Feedback aim to reflect the patient-centred lifeworld of mental health suffering and recovery, including resource dimensions, symptom dimensions, and interpersonal and social role dimensions. Details of Norse Feedback content, implementation and psychometrics are available elsewhere [17, 19, 20, 22, 29, 30, 32, 33].

### Quantitative data analyses

**Fig. 2:**
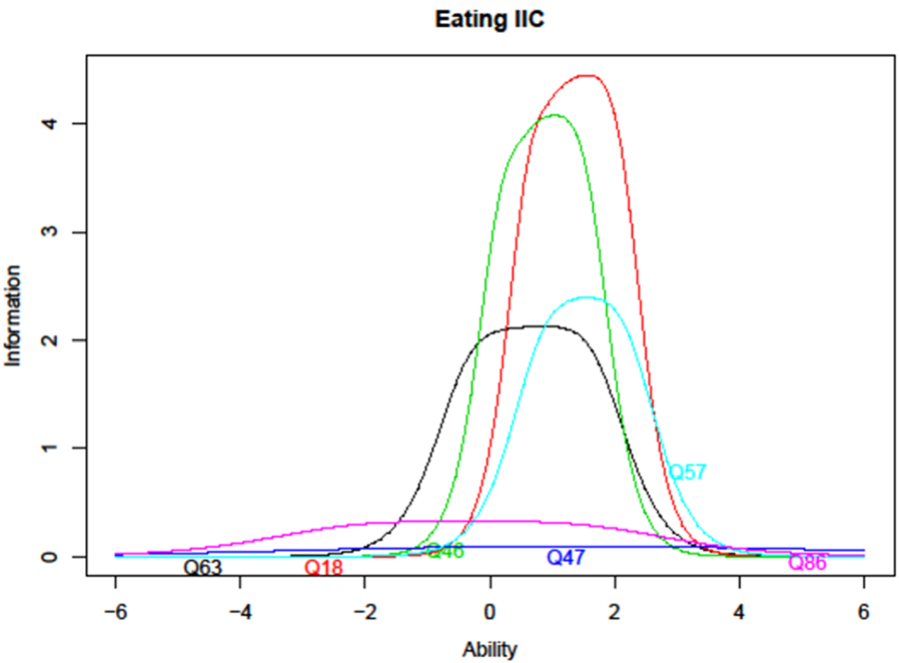
Information curve used for priming interviews

From the first cycle of Norse Feedback implementation, we collected intake reports from 800 patients for analyses. Complete descriptions of the statistical analyses can be found elsewhere [33]. To inform this qualitative study, we presented item information curves that are easily understandable for participants without a statistical background. An item information curve is a visual display of the amount of information an item provides within a scale at each level of severity. As one example of this procedure, Fig. 2 illustrates how focus group discussions of the Eating problems dimension were primed with the item information curves showing that two items (86 and 47) did not contribute to the dimension. Moreover, it showed that the scale had too little information in the difficulty area beyond two standard deviations above population average. When we presented the primer we suggested that this might be a clinically important range for this scale.

### Qualitative data collection

#### Clinician participants

We purposively [34] sampled 10 clinicians to be participants in focus groups [35], a data collection setting particularly suitable for exploring nuances of shared experiences in a group. Inclusion criteria were that clinicians had worked at one of the clinical sites where Norse Feedback had been implemented, and that they had used Norse Feedback across a range of patients. By purposive sampling we aimed to get access to participants with rich information and experiences from different sites. Table 1 provides an overview over the participants’ characteristics.

**Table 1:** Clinician participant characteristics

Participant	Gender	Education	Years of experience	Therapy affiliation
1	Male	Psychiatric nurse	16 years	CBT/MCT
2	Male	Psychiatric nurse	16 years	CBT/MCT
3	Male	Clinical psychologist	13 years	Emotion focused/dynamic
4	Male	Psychiatric nurse	18 years	CBT
5	Woman	Psychiatrist	30 years	CBT
6	Woman	Clinical psychologist	3 years	Emotion focused/dynamic
7	Woman	Psychiatric nurse	25 years	CBT/dynamic
8	Woman	Clinical psychologist	7 years	Emotion focused/dynamic
9	Woman	Clinical psychologist	1 years	CBT
10	Woman	Psychiatric nurse	6	CBT

We conducted three focus groups with the ten clinicians, each lasting two hours, over the course of a two-day meeting. The first focus group was conducted based on an interview schedule developed to explore participants’ general experience with using a clinical feedback system, and two focus groups were structured by the statistical primers (Fig. 2) for all the dimensions to collect participants’ experiences with clinical use and ideas for improvement. All focus groups were audio-recorded and transcribed verbatim for analyses.

#### Patient participants

Inclusion of patients (n = 9) to the study followed a separate logic. When the quantitative analyses showed that we lacked information in some area of the measure, for example as illustrated in Fig. 2 the severe end of the *Eating problems* spectrum, we purposively invited patients with lived experiences with those exact issues. We chose this strategy since patient participants do not have experiences with the CFS across a range of cases, but have particularly rich experiences with their own use and their own core problem. Data collection methods were individual interviews [36] (n = 3) and one focus group [35] (n = 6) to explore which questions or items could provide the needed precision. The interviews were structured by presenting the relevant information curves to the patient and exploring their experience of the included items and their ideas for development. Individual interview duration ranged from 15 to 28 min and the focus group lasted 90 min. Interviews were audio-recorded and transcribed verbatim for qualitative analyses.

CM, MMH and GHM collected the qualitative data in focus groups and individual interviews.

### Qualitative data analysis

Qualitative data was analysed in a stepwise process of reflexive thematic analysis [37, 38]. First, CM, MMH and GHM studied the totality of the data material and discussed how it could inform the research question. Based on these discussions and the authors reflexive notes from the data collection procedure the material was divided into two parts: (a) concrete suggestions on the item level with their corresponding reasons, and (b) experiential accounts, narratives and general ideas. Second, CM made a preliminary thematic structure from stage one’s part b) and presented this back to the group of authors for discussion and refinement. These discussions led to some changes in the thematic focus, in particular with a strengthened focus on the theme of technological user-friendliness that was less pronounced in CM’s preliminary structure. Third, CM checked the resulting themes back with the data to ensure correspondence and find illustrative quotes. After this stage the thematic structure was presented at a scientific conference as work-in-progress to get feedback on its resonance and relevance from colleagues in the field [39]. Fourth, the final thematic structure was written up by the group of authors, with CM taking a lead role. The output of the process is two-fold. We disseminate results in text in this paper, and we saw them in concert with the concrete suggestions from part a) in stage one in making changes from the first to the second implemented version of Norse Feedback in clinical practice.

## Results

From the general discussion of using the system in clinical practice, participating clinicians emphasized that they used the alliance and patient-reported therapeutic needs part of the feedback the most. These parts were easily available and needed in the here-and-now discussions with the patients. When discussing the use of the different dimensions they had varying experiences. Some tracked a smaller selection that they found particularly relevant to their clinical practice and type of patients, and none reported that they used all of the dimensions actively, even if they were open and reported on for an individual patient. In being primed with statistical data about the information inherent in the different dimensions, focus group discussions developed around how this information mirrored their clinical situation, how it could best be represented and if they saw the need for it to be represented at all. In these discussions, aspects that the therapists missed came in the natural flow of the discussion.

Analysing this data material, we found a thematic structure with five dimensions. These were: 1. Added clinical needs, 2. Needs for re-organizing the clinician report, 3.Needs for differentiation of clinical content, 4. User-interface needs, and 5. Item level suggestions.

### Added clinical needs

Participants reported that some of the feedback supported clinical conversations with patients, and that some of the included dimensions did not perform to that end. As one example, substance abuse problems were measured in a way therapists had experienced as insufficiently sensitive to change for the clinical processes that they worked with. They related that many patients had reacted negatively, when both therapist and patient experienced that important work was done and results achieved in the therapeutic process, whereas an honest reply to the items included in this dimension kept the patients with a highly elevated score. They suggested that “*If this is how substance abuse is traditionally measured, we need another scale that measures emerging substance coping experiences, for example not having to avoid shops during the hours they sell alcohol, or being able to go to meetings without drugs”*. The clinical need that they verbalized was support in the process of concretizing changes achieved in the clinical conversation, and they worried that without this need being met, the feedback system could lead to demoralization, both for patient and therapist.

As another example, the dimension for psychotic thinking performed poorly in its psychometric aspects. This came as no surprise to the clinicians, who had found that dimension strange to relate to in their clinical work. They voiced the opinion that confused and paranoid thinking was a matter that was unfitting in a digital self-report system, and that they could not use those pieces of feedback to start constructive dialogues together with their patients. They expressed that “*we need a way in, to start talking about these sensitive matters while normalizing and building safety, not just ask about the worst head on. No one would endorse ‘I am afraid someone will take control over my thoughts’ in a digital self-report—and particularly not those it is true for. If we could take out this dimension, and rather ask about something simple, like forgetfulness or concentration difficulty, then maybe we had the door opened to us and we could take it from there*”. Importantly, to the participant the issue was not what can be measured, but rather how things can be asked in a way that supports clinical conversations directly.

### Needs for re-organizing the clinician report

In the focus groups, one participant related to the other participants that he found the different dimensions to be “*very different things”* and explained that for him, sad affect, or depressed mood as he understood it, was an outcome, that is, something he would expect to start high and end lower as a consequence of constructive therapeutic work—*“but it is not what I work on directly in the sessions”.* Negative rumination, on the other hand, he expressed, he saw as a process in upholding symptoms, and something that was actively shared and worked with in the sessions. *“And in this report you mix it all in the same bowl”,* he ended. This led to an engaged discussion between the participants in the focus group, revising each dimensions for what level they constituted with regard to therapeutic work. From these discussions, participants agreed that dimensions fell within four domains, where one included expressions of symptoms, another included what they considered problem-maintaining processes, another included internal and external resources that the patients had, and the last included dimensions pertaining to patient functioning. One expressed that *“I have patients with very elevated anxiety scores that bite their teeth and have almost no functional impairment, whereas others report only mildly on symptoms dimensions but do not function in their lives at all. These are clinically important matters”.* Participants agreed that the clinical report should be organized to reflect the insights developed through these discussions, aimed at better psycho-pedagogical communication.

Another example that emerged as important under the theme of re-organization was the presentation of suicidality issues. In the version of Norse Feedback that the therapists had used the four items that covered suicidality were presented visually as standardized elevation on a scale. While participants appreciated this as useful for other scales, they discussed how this was particularly unfit for suicidality. *“All my patients will score highly on a scale that joins it together, almost all the time. That is what I work with”,* explained one participant and continued: *“but I know that, that has no extra value to me. What I need to get is to see it the day they suddenly move up a notch or two on the item about fearing to lose control over impulses.”* Through discussing their experiences, participants reached consensus that suicide items needed to be given their separate placeholder in the report, and fed back to clinicians visually as responses to individual items, not as a scale, to fit the way they worked with these issues together with patients.

### Needs for differentiation of clinical content

Discussing the usefulness of the clinical content, participants in the focus groups had different favourite dimensions that they used the most in their clinical work, dependent on the patients they usually worked with and their own therapeutic modality. More often than not, favourite dimensions fell within the domain of *problem maintaining processes* that the participants suggested under the second theme. These were for example avoidance, negative rumination, or demoralization. For these dimensions, that seemed to lie close to different participants’ particular clinical expertise, many shared wishes for further specification and differentiation based on the statistical priming.

For example, the dimension for avoidance had some psychometric issues presented in the primer for development. In the following discussion, clinicians did not merely relate their clinical needs, but offered insights from their expertise. For example, one participant said: “*In my experience, and in the way I work, I think that you get problems here because you seem to put two different processes into the same dimension. They are both avoidance, for sure, but they are not the same. I think the questions that aim at social or people avoidance are very different from those that aim for avoidance of triggers that result in panic-like anxiety. Maybe you put them together for brevity, put I really think you should divide them. At least in two.”* Discussing which “kinds of avoidances” existed and how they related differently to concepts such as “autonomous anxiety dynamics”, “self-image problems” or “trauma” engaged the group. Participants agreed about the need for differentiation, but were not unison about how it should be. They concluded that we needed to test and get responses from the resulting data.

Similar discussions arose in the focus group for dimensions of negative rumination, interpersonal difficulty and resilience/coping, with the same conclusion.

### User-interface needs

An un-scheduled discussion that emerged in the focus groups was how the CFS looked, felt and worked technically. Participants shared frustrations with technical difficulties that had been present in initial phases, and stressed the importance of such a system to “*work without hiccups, all the time”.* Participants related that they often experienced systems in the health organization to prioritize content refinement over user-friendliness, and that they did not think this was wise. One participant expressed: “*Remember what we compete with—what patients are used to. You can download the Facebook app and it looks good, even my 80-year old mother gets how it works. Or another app, for your electricity, it has a lot of information but it really feels easy. They make it like that because they really want to keep you as a customer. That’s the kind of commitment we should aim to communicate to therapists and patients too*”. Participants strongly believed that for their patients it was important that *“hospital systems don’t look like hospital systems”.*

Based on the emergence of this theme we also brought up this issue in the interviews with the patient participants and got insight into experiences that supported the notion that user-interface has great influence on implementation and clinical use.

### Item-level suggestions

As a natural consequence of both the analyses of items’ and dimensions’ psychometric properties, and also following the reported themes of added clinical needs, re-organization- and differentiation needs, a lot of the focus group discussions came to a point where the issue was “*to come up with an item to cover that particular area”.* In several of these discussions, participants could, based on their clinical expertise and experience, come up with specific suggestions on how to formulate the needed items. As such, the focus groups generated a range of potential items that could refine the next iteration in accordance with the discussed needs. However, participants were attentive to the fact that patients would also be purposively invited to the study, with their lived experiences with the phenomena that we wished to develop. Many focus group participants expressed some variation of “*while we can come up with ideas here, the best thing would really be to bring this back to those who have first-hand knowledge*”, manifesting an attitude that people are experts in the experience of their own suffering.

In the interviews and focus group with patients we explored this potential in this study. Generally, being presented with the statistical visual primer and being invited to suggest how we could get more, and more relevant, information into different dimensions, seemed an engaging task for these participants. As one example, when using the primer shown in Fig. 2 in interviews with two patients who were in recovery from eating disorders after having been very ill, the interviewer would ask: *As you can see here, we need help with finding a question that helps us know when things move from very bad to excruciatingly bad. If you can remember from your own experience, the time when you moved from feeling ‘I kind of have some control over this’ to fearing that this might actually not end well at all, what would be a question that would pick up that change in you?”* Interestingly, and of course maybe coincidentally, the two participants came up with similar formulations when exploring their own experiences in the interviews.

Another example that we chose to explore in the focus group format was the reported need to build a dimension that reflected and was sensitive to changes in substance abuse coping. This focus group, consisting of patients in treatment for substance abuse, generated a wealth of items that felt relevant to them in describing the advances made in the initial stage of recovery.

## Discussion

In the present article, we have presented the qualitative results from a mixed-method action research sub-study in the development, refinement and implementation of a clinical feedback system. Many of the participants had been part of defining their needs prior to the development of the CFS [30], and had then used it clinically for 18 months after it was developed. Admittedly, the clinician perspective is dominant in this study, based on the premise that they use the CFS across a range of different patients and thus have a particular generalist experience with its usefulness and shortcomings. The patients’ needs from the system [30] and their experiences with using the system in their own treatment processes have been extensively studied and reported elsewhere [17, 19, 20]. In the present study, they were invited to contribute their lived expertise in particular areas covered by the feedback systems. In terms of practical development of Norse Feedback from cycle one to cycle two, knowledge from all these studies inform the decisions as part of the method. Of particular importance in the results of the present study is how all themes converge around the continuous need to develop the feedback system to mirror and support the clinical processes as they are practiced.

Obviously, the reported results in this study have their immediate relevance for the Norse Feedback action research program, and to warrant dissemination to a wider readership, relevance for other contexts and other clinical feedback systems should be discussed. Many prominent and well-documented feedback systems exist, aiming to contribute to improved mental health services and clinical outcomes in their respective catchment areas. Some important implications of this study seems worth mentioning.

### Implementation science

Difficulties with implementing ROM/CFSs in ordinary practice are recognised and discussed across mental health contexts [7, 40], and they oftentimes take the form of explicit and implicit opposition from end-users [41]. This seems very important, as studies show that therapists attitudes to using feedback influence patients’ outcomes [42, 43] and that the non-use of feedback that is collected can be very burdening for the patient [19]. In consequence, the most important costs of a failed CFS implementation might not be the ill-spent resources for the organization, but the health cost for the patient receiving something worse than he or she would have received without the implementation in the first place. To solve this dilemma, we often turn to implementation science [7, 40].

Although implementation science has developed multifaceted intervention strategies to account for complexity [44], much used concepts remaining in use are still *barriers* and *facilitators* [45]. Coming from a context where objectively better medicines or medical procedures would improve health, barriers to uptake needed to be found, fought and overcome. The idea is straight-forward, but does it fit in the context where complex change (systematically collecting and using patient reported data for all patients) is to happen in a complex setting (the interpersonal therapy process between two people) to achieve better outcomes for some (but not all [3, 8]) patients?

Considering the results in this paper, many of them would conceivably be conceptualized as *barriers* to uptake in an implementation study, if the basic premise were that the CFS in itself could not change. For example, in the first theme about added clinical needs, participants essentially report that the CFS cannot do *this* for me, and in the third theme, they report that *this* does not reflect my clinical reality particularly well. Perceived failures of CFSs to capture the necessary complexity of mental health is a described barrier to acceptance in multiple studies [18]. However, in the context of a participatory action research project such worries get another meaning. Rather than barriers, they become potentials for voicing your feedback and contributing to a better CFS. One implication of the present study is that PRNs [26] and participatory action research [28] can facilitate processes of implementation and development via engagement and dialogue. This might be relevant for CFSs aiming to develop to provide better services. Arguably, what is traditionally seen as barriers to uptake can also be seen as legitimate and informed opposition to shortcomings.

## Limitations

This is a qualitative study of Norse Feedback, implemented by researchers who are all involved in the Norse Feedback project. This suggests that one should be mindful of potential biases in data collection and analyses. The group of researchers involved in the project works to address this potential bias by holding an attitude of critical self-evaluation at the front of our work. Evidently, the risk for bias can be seen as reduced by the scope of the paper, which is to explore how the CFS needs to develop to become better suited to clinical needs rather than to document that it is good as is. Moreover, Norse Feedback has been used by thousands of patients and more than one hundreds of therapists, out of which a small sample of participants have been invited to this study. This suggests that one should be careful in generalizing the results of the current study across different contexts, both within and beyond the Norse Feedback catchment area.

## Conclusions

We studied clinicians’ and patients’ experiences with using a clinical feedback system over a period of 18 months, and found that they could contribute to developing the system by adding clinical needs, needing re-organization of the clinical report, differentiating clinical constructs measured by the CFS and suggest new items to aid the development of the constructs.

## Data Availability

Data materials are available for reasonable request during the project period. Data will be deleted after the project period.
